# Epistaxis: the cause found beyond the nose

**DOI:** 10.4322/acr.2020.207

**Published:** 2020-12-08

**Authors:** Rahul Verma, Vinay Nagaraja Gowda, Ashok Singh, Madhu Priya, Sanjeev Kishore, Prashant Pranesh Joshi

**Affiliations:** 1 All Institute of Medical Science Rishikesh, Department of Pathology. Rishikesh, Uttarakhand, India; 2 All Institute of Medical Science Rishikesh, Department of Ear, Nose and Throat. Rishikesh, Uttarakhand, India

**Keywords:** Carcinoma, Renal Cell, Epistaxis, Paranasal Sinuses

## Abstract

Renal cell carcinoma (RCC) is a malignant disease that is often diagnosed at a metastatic stage. The head and neck represent up to 3% of the metastatic RCC, and the paranasal sinus area is one of the least involved sites. Here, we introduce the case of a 74-year-old female patient who presented with a history of traumatic nasal bleed. A cranial computed tomography scan and magnetic resonance imaging showed a fronto-ethmoidal mass with pachymeningeal involvement. A nasal biopsy from the paranasal sinuses was taken. On histopathological examination, metastatic clear cell carcinoma was the main hypothesis, which later was confirmed to be RCC on immunohistochemistry. On further radiological examination, an exophytic mass was depicted in the kidney’s upper and middle pole. The patient had no renal complaints and was asymptomatic. Fronto-ethmoidal sinus is a rare site for metastatic RCC, especially in cases where the patient is asymptomatic. Early detection by keeping RCC metastasis as the differential diagnosis in such cases can lead to early treatment and improve the overall survival of the patient.

## INTRODUCTION

Renal cell carcinoma (RCC) accounts for 90% of all renal malignancies^1^ and is more common among middle-aged and elderly men.^2^ The most common sites are the lung (76%), regional lymph nodes (66%), bone (42%), and liver (41%).^3^ Paranasal sinuses are a rare site for RCC to metastasize.^4^ Thus, metastatic carcinoma to the paranasal sinuses of the primary site in the kidney is a rare diagnosis that should be raised in asymptomatic middle-aged patients presenting with a unilateral paranasal sinus mass and epistaxis. We report the case of fronto-ethmoidal sinus involvement by metastatic RCC in an asymptomatic and undiagnosed RCC patient.

## CASE REPORT

**Figure 1:**
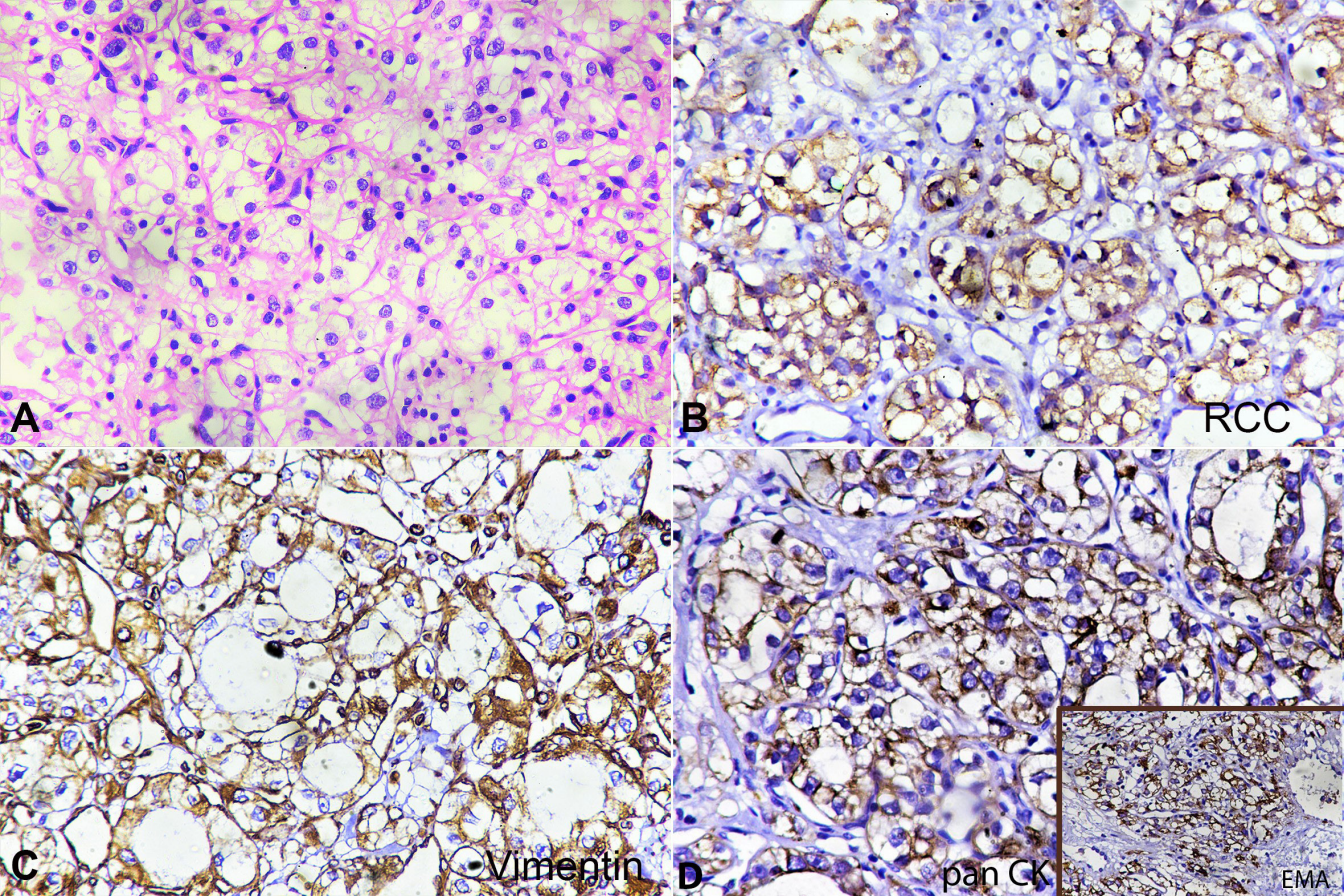
**A –** A tumor comprised of clear cells arranged in clusters. The cells have abundant clear-to-vacuolated cytoplasm and a central round nucleus showing mild pleomorphism. Nucleoli are inconspicuous (H&E 400X). **B –** Tumor cells showing strong membranous staining with RCC antigen (400X). **C –** Tumor cells showing strong membranous staining with vimentin. **D –** Tumor cells showing no staining with SMA (400X). RCC = renal cell carcinoma; SMA = smooth muscle actin.

A 74-year-old female patient presented with complaints of nasal bleeding after a road traffic accident. On clinical examination, a growth in the nasal cavity was visualized using a zero-degree endoscope. Contrast-enhanced magnetic resonance imaging depicted irregular soft tissue in the frontal and ethmoid sinuses projecting into the left nasal cavity, and the left orbital apex. A breach of the frontal sinus boney walls and intracranial extension with thickening of the pachymeninges was also noted. A punch biopsy was undertaken from the left intranasal growth under general anesthesia after decongesting the nasal cavity with adrenaline and saline (1:4). The biopsy was sent for histopathological examination, which revealed a neoplasia composed of clear vacuolated cells arranged in sheets, cords, and trabeculae, highly vascular, with numerous interspersed thin-walled blood vessels. No distinct cell borders, cell membrane thickening, or any raisin-like nuclei were seen (Figure 1A). On immunohistochemical examination, the tumor cells were strongly positive for RCC antigen (Figure 1B), vimentin (Figure 1C), panCK and EMA (Figure 1D), but showed negativity for smooth muscle actin, CD34, CD 31, CD10, HMB-45, and S-100, PAX-8 and CK 7.

Based on the patient’s morphology and immunohistochemistry, the possibility of metastatic RCC was indicated. On further evaluation, the computed tomography (CT) urography revealed an exophytic and necrotic lesion measuring 7 cm in its longest axis over the upper and mid pole of the left kidney. Also, the imaging study showed tumor infiltration of the left renal vein with a thrombus formation. Meanwhile, the patient had an episode of massive epistaxis followed by a myocardial infarction (MI), but with a good outcome. After clinical stabilization, the patient was referred to medical oncology and was started on pazopanib 800 mg once daily for 30 days. Currently, she is doing well and is asymptomatic.

## DISCUSSION

RCC is most commonly seen in the age group of 30–60 years and is routinely present with flank pain, palpable mass, and gross hematuria.^5^ It shows a variable growth rate, and metastases may be found anywhere in the body. The most common sites are the lung (76%), regional lymph nodes (66%), bone (42%), and liver (41%).^3^ RCC metastasis to the head and neck—specifically in the paranasal sinuses—is rare.^6^ ^,^ ^7^ Only 100 cases have been reported so far to our knowledge. In these cases, ethmoid sinus (50%) was the most common site for metastasis, followed by maxillary sinus (39%), sphenoid sinus (19%), and frontal sinus. In 21 cases, the diagnosis of metastasis was made before the primary tumor was diagnosed.^6^ The most common route for distant metastasis is by the bloodstream, especially through the renal vein.^8^ The maxillary sinuses are more frequently affected by the RCC metastatic tumor, followed by the ethmoid sinus, frontal and sphenoid.^9^ The paranasal sinuses are a rare location of metastasis; however, the most frequent primary tumor to metastasize to paranasal sinuses is RCC.^2^ Tumors great than 3 cm are more prone to cause metastasis and microvascular thrombi formation, which may lead to increased incidence of MI.^10^

As our patient was stable with no cardiovascular impairment, the episode of MI could have been due to atherosclerosis considering her age; however, in view of the distant metastasis to the paranasal sinuses and the size of the tumor, tumor thromboembolism should be ruled out. RCC with paranasal sinus metastasis as the first presenting sign is rare. Clinically, RCC metastasis to the fronto-ethmoidal sinuses may present with nasal bleeding; this raised the suspicion in our case along with the mass.^11^ A concomitant history of trauma may lead to the wrong approach; therefore, detailed clinical and histopathological examinations were of vital importance. Histological features should be complemented with the immunohistochemical markers. RCC is positive for panCK, RCC antigen, and vimentin. CK7 is positive in papillary renal cell carcinoma but negative in clear cell carcinoma^12^ In the case of metastatic RCC it is crucial to confirm the renal origin of the tumor by using PAX-8. In our case, tumor cells’ morphology favored clear cell carcinoma with strong immunopositivity for RCC antigen, vimentin, panCK, and EMA. Tumor cells were negative for CK7, thus ruling out the papillary renal cell carcinoma. In our case PAX-8 lost its expression at the metastatic site, and thus was negative. Although PAX-8 expression is frequently positive in most of the clear cell RCC, loss of its expression does not rule out the renal origin of the tumor.^13^ Thus, in such cases, the morphology as well as immunohistochemical findings, should be contemplated with the clinic-radiological findings.

## CONCLUSION

RCC metastasis presenting as epistaxis is rare, and a detailed examination via urinalysis, intravenous pyelogram, and CT is required in case of suspicion. In these cases, epistaxis is usually present. The precise diagnosis will always depend on the histopathological examination along with ancillary techniques.
